# 

**Published:** 1992-03

**Authors:** 


Volume 107(i) March 1992

What's New from The British Journal of Surgery
Ethics in Transplantation
Survey of Services to Patients in General Practice
An Audit of Barium Meal Examinations in General Practice
Obituary — Henry Dendy Moore (1912-1992)
Correspondence
From our Correspondents
Crossword 1
What is this Life if Full of Care
Book Reviews
South West Paediatric Club
The Place for Paediatricians
Wessex Branch of The Association of Clinical Pathologists
South West Radiologists Association

NATIONAL LIBRARY
JAN 29 1993
OF MEDICINE

FORMERLY BRISTOL MEDICO-CHIRURGICAL JOURNAL

ISSN: 0960 6440 NLM Code B5Q

